# EphA2 Promotes the Development of Cervical Cancer through the CXCL11/PD-L1 Pathway

**DOI:** 10.1155/2022/4886907

**Published:** 2022-11-28

**Authors:** Xinyue Zhao, Jiaxi Liu, Dongdong Jin, Chenchen Ren, Li Yang, Yuanhang Zhu, Changhao Huang, Leilei Ding, Zimeng Wu, Ke Shen, Zhen'an Zhang, Huanhuan Chen, Nannan Wang

**Affiliations:** ^1^Department of Obstetrics and Gynecology, The Third Affiliated Hospital of Zhengzhou University, Zhengzhou, China; ^2^Zhengzhou Key Laboratory of Cervical Disease, The Third Affiliated Hospital of Zhengzhou University, Zhengzhou, China; ^3^National Clinical Research Center for Obstetrics and Gynecology, Henan Branch, The Third Affiliated Hospital of Zhengzhou University, Zhengzhou, China; ^4^Organ Transplant Center, Xiangya Hospital, Central South University, Changsha, China

## Abstract

Erythropoietin-producing hepatoma receptor A2 (EphA2), receptor tyrosine kinase, the most widespread member of the largest receptor tyrosine kinase family, plays a critical role in physiological and pathological conditions. In recent years, the role of EphA2 in the occurrence and development of cancer has become a research hotspot and is considered a promising potential target. Our previous studies have shown that EphA2 has an indisputable cancer-promoting role in cervical cancer, but its related mechanism requires further research. In this study, high-throughput sequencing was performed on EphA2 knockdown cervical cancer cells and the control group. An analysis of differentially expressed genes revealed that EphA2 may exert its cancer-promoting effect through C-X-C motif chemokine ligand 11 (CXCL11). In addition, we found that EphA2 could further regulate programmed cell death ligand 1 (PD-L1) through CXCL11. This has also been further demonstrated in in vivo experiments. Our study demonstrated that EphA2 plays a tumor-promoting role in cervical carcinoma through the CXCL11/PD-L1 pathway, providing new guidance for the targeted therapy and combination therapy of cervical carcinoma.

## 1. Introduction

Cervical carcinoma is the most common gynecological malignancy with high morbidity and mortality [1]. Worldwide, the incidence of cervical carcinoma is increasing every year [2]. There are more than estimated 500,000 new cases of cervical carcinoma worldwide each year, with more than 300,000 deaths [3]. Although vaccination against human papillomavirus and cervical carcinoma screening can effectively prevent the occurrence of cervical carcinoma and greatly decrease the incidence of cervical cancer [4], owing to various obstacles in the implementation process, the overall preventive effect achieved by these methods is not ideal, especially in some countries and regions with heavier burdens of disease [5]. Data show that most patients are diagnosed with advanced cervical cancer [6]. For patients with advanced cancer and recurrent and metastatic disease, existing treatments have limited efficacy and nonnegligible toxic effects, which make the prognosis poor. It remains an urgent and important task to investigate the specific molecular mechanisms of cervical carcinoma occurrence and development and provide more different options for related treatment.

EphA2 was first discovered in 1990 in the screening of the human epithelial (HeLa) complementary DNA (cDNA) library and called epithelial cell kinase. It is expressed in the cell membranes of most epithelial tissues [7]. EphA2 is expressed at high levels in various solid tumors [8–10]. It is often closely associated with a poor prognosis, increased invasiveness, and decreased survival. In 2004, Wu et al. discovered that a high level of EphA2 protein expression in cervical cancer could decrease overall survival, suggesting that EphA2 may be a valuable clinical marker for cervical cancer [11]. Our previous results have confirmed that EphA2 is highly expressed in cervical carcinoma and tumorigenic both in vitro and in vivo. Relevant experimental results showed that the expression level of EphA2 in cervical carcinoma was positively correlated with the malignant biological behavior ability of tumor cells and negatively correlated with an overall survival rate of patients [12]. Although the cancer-promoting effect of EphA2 in cervical cancer has been confirmed, the related mechanism of its promoting effect in cervical carcinoma needs to be studied further. This study will be important for full understanding of the function of EphA2 in cancer malignancy and is also conducive to developing new treatment strategies for cervical cancer.

At the beginning of this study, we performed an RNA sequencing analysis on EphA2 knockdown cervical cancer cells and controls. By analyzing the sequencing data, we found that the expression level of the differentially expressed gene CXCL11 decreased with the decrease of EphA2 expression. CXCL11 (also known as I-TAC) belongs to the chemokine superfamily and is expressed at a high level in different solid tumors, promoting tumor growth, metastasis, and lymphocyte infiltration [13–16]. Our experimental results show that EphA2 promotes the proliferation, invasion, and metastasis of cervical carcinoma through CXCL11. Additionally, we found that PD-L1 (also referred to as CD274) might mediate the cancer-promoting effect of EphA2-CXCL11 in cervical cancer. Experimental results showed that the addition of CXCL11 to the EphA2 knockdown SiHa cell line recovered the decreased PD-L1 expression. We speculate that the targeting EphA2 and PD-L1 may produce more significant tumor suppression than monotherapy. That is, targeting EphA2 may improve the sensitivity of cervical cancer to PD-L1-targeted therapy. For patients who are insensitive or resistant to anti-PD-1/PD-L1 therapy, simultaneously targeting EphA2 may be a new therapeutic strategy. Of course, this requires further verification.

This study is the first to propose that EphA2 plays a role in promoting cancer through the CXCL11/PD-L1 pathway in cervical cancer, which opens up new possibilities for targeted therapy and combined therapy of cervical carcinoma.

## 2. Materials and Methods

### 2.1. Cell Culture

The cervical cancer cell line SiHa was donated by the First Affiliated Hospital of Zhengzhou University. Other cell lines (HeLa, Caski, H8, and 293T cell linea) used in this experiment were borrowed from related research groups in our laboratory. Cells were grown in Dulbecco's Modified Eagle's Medium (Solarbio, Beijing, China) containing 10% fetal bovine serum (ThermoFisher Scientific, Shanghai, China) and 1% penicillin-streptomycin double antibody (Beyotime, Shanghai, China) in a 37°C incubator with 5% CO_2_.

### 2.2. Knockdown of EphA2 by Small Interfering RNA Transfection and RNA Sequencing

A certain amount of SiHa cells were seeded in six-well plates so that the cell density reached from 30% to 50% after 1 day, and then, transfection was performed. EphA2 knockdown small interfering RNA (sense 5′-UGACAUGCCGAUCUACAUGTT-3′; antisense 5′-CAUGUAGAUCGGCAUGUCATT-3′) and its negative control were ordered from GenePharma (Shanghai, China). Transient transfection was performed using Lipo6000 Transfection Reagent (Beyotime) according to the product instructions, and changes in the expression level of EphA2 in SiHa were detected by quantitative real-time polymerase chain reaction (qRT-PCR). After it was verified that EphA2 was indeed knocked down, the RNA of the transfected cells was extracted and analyzed by RNA sequencing together with the control group. Sequencing technology was provided by OKEx (Shanghai, China). The relevant sequencing results have been submitted to the National Center for Biotechnology Information Sequence Read Archive (BioProject ID: PRJNA853109).

### 2.3. Bioinformatics Analysis

RNA sequencing results of tumor and control samples from TCGA and GTEx databases were analyzed by GEPIA (https://gepia.cancer-pku.cn/#analysis). Transcript per million values were calculated, and the expression levels of genes were presented using the log 2 (transcript per million + 1) scale. The cutoff for significance was chosen at a *p* value of 0.01. In this article, we analyzed the differential expression of CXCL11 in 306 cervical carcinoma samples and 13 normal cervical samples and the correlation between CXCL11 and PD-L1 in cervical cancer by GEPIA.

### 2.4. RNA Extraction and qRT-PCR Analysis

Total RNA was extracted from cells by RNAiso Plus (Takara, Beijing, China) following the procedure described in the instructions. Subsequently, 900 ng of total RNA was reverse transcribed into cDNA using an UEIris II RT-PCR System for First-Strand cDNA Synthesis (with dsDNase) (US Everbright, Suzhou, China) kit. The obtained cDNA was subjected to qRT-PCR by the ABI 7500 real-time PCR system (Applied Biosystems, Foster City, CA, USA) using the Hieff qPCR SYBR Green Master Mix (Yeasen, Shanghai, China) kit. Using the glyceraldehyde-3-phosphate dehydrogenase (GAPDH) gene as an internal reference, the relevant experimental data were analyzed by the 2^−ΔΔCT^ method [17]. The primer sequences involved in this experiment are shown in Table 1, and all primers were from ShangYa (Zhengzhou, China).

### 2.5. Protein Extraction and Western Blot Analysis

Total cellular proteins in six-well plates were extracted using high-efficiency RIPA cell lysis buffer (Solarbio, Beijing, China). After removing the medium and washing with phosphate-buffered saline, 150 *μ*L of lysis buffer was added to each well. The six-well plates were lysed on ice for 30 minutes. When the lysis is complete, the lysis mixture was pipetted into a centrifuge tube. The supernatant containing total cellular protein was collected after centrifugation. The extracted protein concentration was calculated by the bicinchoninic acid assay kit (Biomed, Beijing, China) according to the reagent instructions.

By electrophoresis, proteins were separated on sodium dodecyl sulfate-polyacrylamide gel electrophoresis at the corresponding concentrations. The proteins were then transferred from the gel to polyvinylidene fluoride membranes (Millipore, Billerica, MA). After washing off the transfer fluid, the membrane was blocked in 5% skim milk for 2 hours. After blocking, they were incubated with the corresponding primary antibody (Table 2) overnight at 4°C. GAPDH was used as an endogenous control. The primary antibody was then washed off, and the membrane was incubated in the secondary antibody for 1 hour at room temperature on a shaker. After eluting the secondary antibody, mix solution A and solution B in Super ECL prime (US Everbright, Suzhou, China) 1 : 1 according to the reagent instructions. The mixed developer was dropped onto the polyvinylidene fluoride membrane, and the immunoreactive protein bands were detected using a GelView 6000 plus Intelligent Image Workstation (Guangzhou Biolight Biotechnology Co., Ltd., Guangzhou, China).

### 2.6. CXCL11 Enzyme-Linked Immunosorbent Assay

The cell culture supernatants of the EphA2 knockdown group and the control group were collected and purified by centrifugation at 1000*g* for 20 minutes to remove impurities and cell debris. After removing impurities and cell debris, the content of CXCL11 protein in the samples was measured by the human CXCL11 enzyme-linked immunosorbent assay kit (Elabscience Biotechnology, Wuhan, China) following the steps described in the reagent instructions.

### 2.7. Vector Construction, Cell Transfection, and Establishment of Stable Cell Line

To establish stable EphA2 knockdown SiHa cells, we ordered the short hairpin RNA clone RNA interference plasmid (vector name: GV248) for human EphA2 and the corresponding negative control at GeneChem (Shanghai, China). The interfering plasmid and the accessory plasmid were cotransfected into 293T cells by using the JetPrime transfection reagent (Polyplus transfection, Illkirch, France). Virus-containing cell supernatants were collected at 48 hours and 72 hours after transfection, respectively.

A certain amount of SiHa cells was seeded in six-well plates the day before the supernatant was collected so that the cell density was 20–30% when the virus-containing supernatant was collected. The collected virus-containing cell supernatant was purified by centrifugation and added to a six-well plate to infect SiHa cells. Stably transfected cell lines were then screened with 2 *μ*g/mL puromycin, and the EphA2 knockdown effect was verified by qRT-PCR and Western blot analysis. The stably transfected strains obtained by screening will be used for subsequent cell experiments.

### 2.8. Cell Counting Kit-8 Assay

Cells were seeded into 96-well plates at a density to ensure that each well contained 2 × 10^3^ cells. The experimental group was incubated with 100 *μ*L of complete medium containing different concentrations (0, 5, 25, 50, and 100 ng/mL) of CXCL11 (300-46, PeproTech, Rocky Hill, NJ), whereas the blank control group was added with an equal volume of complete medium. The 96-well plate was placed in a cell incubator and incubated between 24 and 48 hours. At the corresponding time point, the medium or the mixture of medium and CXCL11 in the wells to be tested was first replaced with a new medium to remove the possible effect of the drug on the absorbance. Then, 10 *μ*L of CCK8 solution (US Everbright) was added to the wells to be tested in the dark. After adding Cell Counting Kit-8, we put the 96-well plate back into the 37°C incubator for 2.5 hours, and finally, measure the optical density value at 450 nm using a microplate reader (ThermoFisher Scientific).

### 2.9. Transwell Migration and Invasion Assay

For migration experiments, cells need to be resuspended in a serum-free medium, and the cell concentration was adjusted after cell counting so that the cell concentration was 5 × 10^5^ cells/mL. We added 100 *μ*L of the cell suspension to each upper chamber, and 600 *μ*L of culture medium containing 20% fetal bovine serum and corresponding concentrations of CXCL11 (0 and 100 ng/mL) were added to the lower chamber.

After 24 hours incubation in the incubator, the medium in the upper chamber was discarded, and the pierced cells were fixed with 4% paraformaldehyde and then stained with 0.1% crystal violet solution. After staining, the chamber was washed with phosphate-buffered saline, counted, and photographed under a light microscope. For invasion experiments, 100 *μ*L of the cell suspension was also added to the upper transwell chamber, but Matrigel matrix (Corning, Cambridge, MA) was overlaid on the bottom of the upper chamber before adding the cells. The rest of the steps are the same as in the migration experiment.

### 2.10. Wound Healing Assay

Cells were evenly seeded in six-well plates the day before scratching, resulting in nearly 100% cell confluency after overnight culture. We used the tip of a 20 *μ*L pipette tip to draw a straight line across the confluent monolayer of cells. The streaked cells were washed off with phosphate-buffered saline, and 2 mL of fresh serum-free medium containing the corresponding concentrations of CXCL11 (0 and 100 ng/mL) were added to each well. Then, we put the six-well plate back into the incubator to continue culturing, and the scratches were observed and photographed under a microscope at 0, 12, 24, 36, and 48 hours. The scratch area was calculated and analyzed with Image J software (NIH, Bethesda, MD).

### 2.11. Establishment of the Subcutaneous Transplanted Tumor Model

Female BALB/c-nu mice (5 weeks) (Spelfer Biotechnology, Beijing, China) were reared in the specific pathogen-free rearing room of the Animal Experiment Center of Zhengzhou University. Nude mice were acclimated to the rearing environment for 1 week, and then, the mice were randomly divided into two groups: the sh-EphA2 group and the sh-NC group. Then, EphA2 stably transfected and control SiHa cells in the logarithmic growth phase were collected and suspended in the serum-free medium, and the cell concentration was adjusted to 2 × 10^6^ cells/mL. Cells (100 *μ*L/mouse) were injected subcutaneously into the middle and posterior parts of the right armpit of nude mice to establish a subcutaneous xenograft tumor model in nude mice. Xenograft tumors were measured with vernier calipers and the tumor volume was calculated using the formula (width^2^ ×  length)/2. Measurements were taken weekly after injection and every 3 days after 2 weeks. Five weeks after injection, mice were humanely killed by cervical dislocation after inhalation of isoflurane. Tumors were removed, blindly weighed, and snap-frozen in liquid nitrogen for subsequent Western blot analysis. All animal protocols in this study were approved by the Animal Research Ethics Committee of Zhengzhou University (ethical approval number: 202204010101) and followed the “Guidelines for the Care and Use of Laboratory Animals.”

### 2.12. Data Analysis and Statistics

All experiments in this study were independently repeated no less than three times, and all data were analyzed by GraphPad Prism 9.0 software. The results were expressed as mean ± standard deviation. The statistical significance was assessed using the one-way analysis of variance and Tukey's multiple comparison test or *t*-test. A *p* value of less than 0.05 was considered statistically significant (^*∗*^*p* < 0.05; ^*∗∗*^*p* < 0.01; ^*∗∗∗*^*p* < 0.001; ^*∗∗∗∗*^*p* < 0.0001).

## 3. Results

### 3.1. EphA2 Knockdown Generates Differentially Expressed Genes

**Figure 1:**
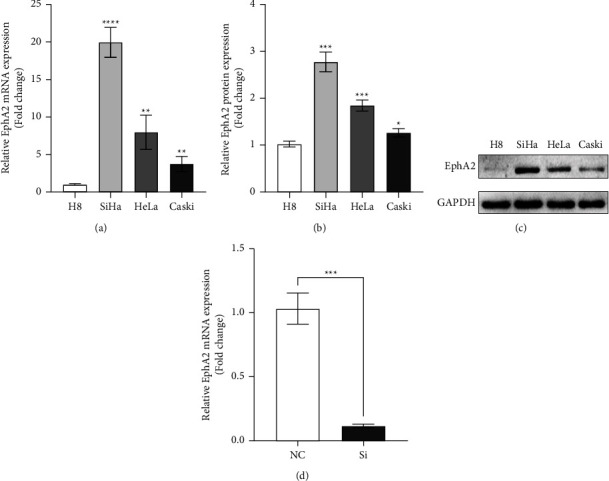
Establishment of EphA2 knockdown cells using the cervical cancer cell line. EphA2 messenger RNA expression levels (a) and protein expression levels (b) in (c) cervical cancer cell lines (SiHa, HeLa, and Caski) and H8 human normal cervical epithelial cell line. (d) Relative protein expression of EphA2 in SiHa after small interfering RNA transfection. ^*∗*^*p* < 0.05; ^*∗∗*^*p* < 0.01; ^*∗∗∗*^*p* < 0.001.

First, we detected the expression level of EphA2 in various cervical cancer cells, including Caski, HeLa, SiHa, and normal human cervical epithelial cells by qRT-PCR and Western blot analysis. The data obtained showed that the messenger RNA and protein levels of EphA2 in three cervical cancer cells were higher than those in normal human cervical epithelial H8 cells, and the expression level of EphA2 in SiHa was significantly higher than that in other cells (Figures 1(a)–1(c)). To explore the molecular mechanism of EphA2's tumor-promoting effect in cervical cancer, we transfected SiHa cells with EphA2 knockdown small interfering RNA. The knockdown effect of EphA2 was verified by qRT-PCR (Figure 1(d)).

**Figure 2:**
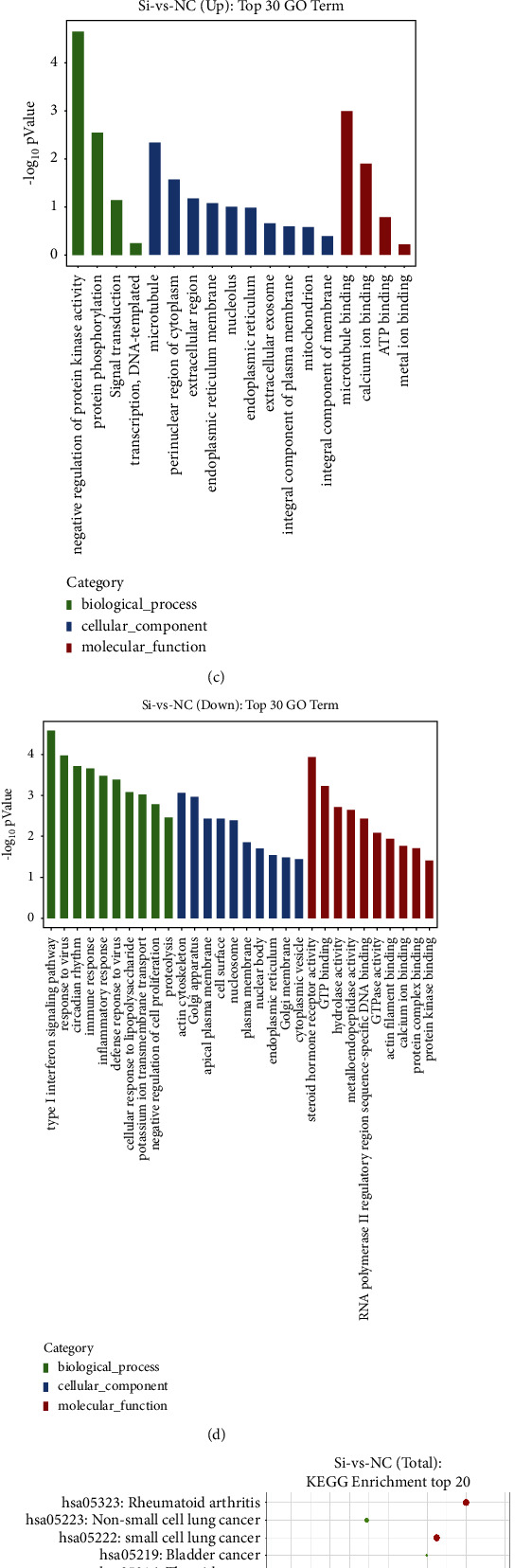
Differentially expressed genes produced by the EphA2 knockdown. (a) Volcano map reflecting the expression of differential genes. Gray is the gene with an insignificant difference, and red and green are the genes with significant differences. (b) Cluster analysis of differential gene expression levels. Red indicates a relatively high expression of protein-coding genes, whereas blue indicates a relatively low expression of protein-coding genes. (c)-(d) GO enrichment map of downregulated and upregulated genes. The horizontal axis is the GO entry name, and the vertical axis is −log10 *p* value. (e)-(f) Bubble plot of KEGG enrichment analysis of differentially expressed genes. The horizontal axis enrichment score is the enrichment score. The larger the bubble, the more the number of differential protein-coding genes contained in the entry. The bubble color changes from purple-blue-green-red. The smaller the enrichment *p* value, the more significant the degree is.

By sequencing the RNA of the knockdown group and the control group, we obtained a clean data of 38.16 G. We aligned the reads to the reference genome and analyzed the expression of protein-coding genes in different samples based on the alignment results. Through differential screening, 211 differentially expressed genes were finally obtained (Figure 2(a)), including 47 upregulated genes and 164 downregulated genes. The clustering analysis results of a differential grouping of each group are shown in Figure 2(b), where red indicates relatively highly expressed protein-coding genes and blue indicates relatively lowly expressed protein-coding genes. After obtaining the differentially expressed genes, we performed gene ontology (GO) and KEGG enrichment analyses on the differentially expressed genes. The GO analysis of upregulated genes was mainly enriched in biological processes, such as negative regulation of protein kinase activity, protein phosphorylation, and microtubule binding (Figure 2(c)). The downregulated genes are mainly involved in the type I interferon signaling pathway, immune response, inflammatory response, and so on (Figure 2(d)). The top 20 of KEGG enrichment results of differentially expressed genes are shown in Figure 2(e). The downregulated genes were mainly enriched in cytokine-cytokine receptor interaction, the Jak-STAT signaling pathway, the NOD-like receptor signaling pathway, the Toll-like receptor signaling pathway, and among others (Figure 2(f)).

### 3.2. EphA2 Targets the Expression of CXCL11 in Cervical Cancer

**Figure 3:**
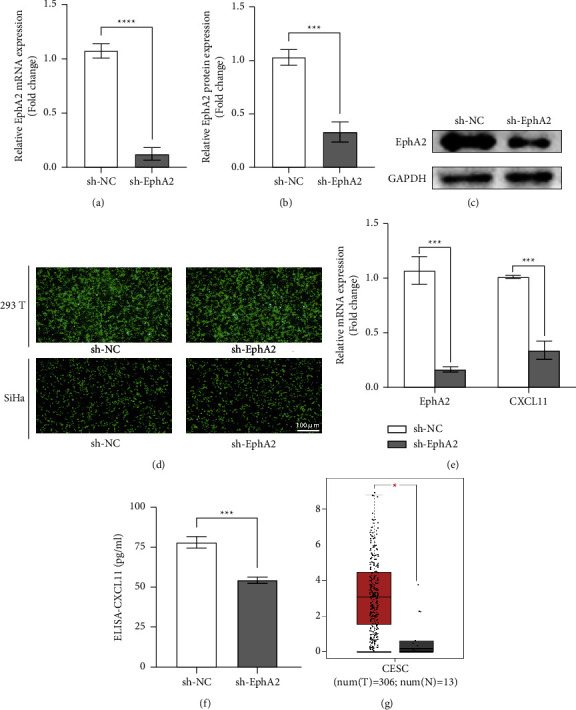
EphA2 regulates the expression of CXCL11 in cervical cancer. (a) Relative messenger RNA expression of EphA2 in SiHa after transfection. (b)-(c) Relative expression and gray value of EphA2 protein band in SiHa after transfection. (d) Green fluorescence indicates transfection efficiency greater than 80%. (e) Relative messenger RNA expression of EphA2 and CXCL11 in SiHa after transfection. (f) Relative expression of autocrine CXCL11 after transfection. (g) Expression of CXCL11 in cervical cancer and normal cervical tissue in GEPIA database.^*∗*^*p* < 0.05; ^*∗∗*^*p* < 0.01; ^*∗∗∗*^*p* < 0.001.

To search for the downstream molecular mechanism of the cancer-promoting effect of EphA2, we carefully analyzed the meaningful differentially expressed genes in the sequencing results. Combined with the KEGG enrichment results, the regulatory effect of EphA2 on CXCL11 was elucidated. This finding is consistent with existing research, which has now confirmed that CXCL11 is involved in the paracrine signaling that promotes immune activation and autocrine signaling that promotes tumor cell proliferation and metastasis [18]. Through the autocrine production of CXCL11, tumor cells can regulate their own directional movements and influence processes such as cancer cell proliferation, angiogenesis, and immune evasion [19]. Thus, we used the viral supernatant produced by the infection of 293T cells with the packaging plasmid to infect SiHa cells and established a stable EphA2 knockdown strain (sh-EphA2). The EphA2 knockdown efficiency in SiHA cells was validated by qRT-PCR, Western blotting (Figures 3(a)–3(c)), and green fluorescent protein (Figure 3(d)).

Subsequently, we verified the regulatory effect of EphA2 on CXCL11 suggested by the sequencing results by qRT-PCR and enzyme-linked immunosorbent assay. The experimental results indicated that EphA2 may play a role in promoting cancer through CXCL11 in cervical cancer (Figures 3(e) and 3(f)). The results of the tumor/normal differential expression analysis provided by the GEPIA database also supported this speculation (Figure 3(g)).

### 3.3. CXCL11 Rescued the Suppression of Proliferative Capacity of Cervical Cancer Cells by the EphA2 Knockout

**Figure 4:**
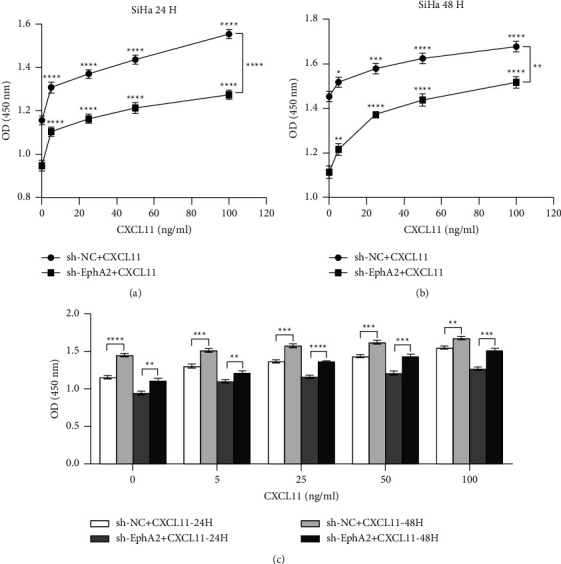
Exogenous supplementation of CXCL11 rescued the inhibitory effect of EphA2 on proliferative capacity in cervical cancer. (a), (b) CCK8 assay for cell viability of cervical cancer cells treated with different concentrations of CXCL11 between 24 and 48 hours to evaluate the effect of CXCL11 on the proliferative ability of cervical cancer cells after the EphA2 knockdown. (c) The effect of CXCL11 on the proliferation of cervical cancer cells is time and concentration-dependent.^*∗*^*p* < 0.05; ^*∗∗*^*p* < 0.01; ^*∗∗∗*^*p* < 0.001.

To further demonstrate that CXCL11 mediates the cancer-promoting effect of EphA2 in cervical carcinoma, we analyzed the effect of CXCL11 on the proliferation ability of the sh-EphA2-SiHa cell line by the CCK8 assay. Data from the CCK8 assay demonstrated that the knockdown of EphA2 led to the weakening of the proliferative capacity of cervical cancer cells, whereas the replenishment of CXCL11 leads to a significant recovery of the proliferative capacity of sh-EphA2-SiHa cells. In addition, we found that CXCL11 also enhanced the proliferative capacity of control SiHa cells. CXCL11 seems to be concentration-dependent in enhancing the ability of cells to proliferate (Figures 4(a)–4(c)). These results suggest that CXCL11 may mediate the enhancement of the ability of EphA2 to proliferate and clone in cervical cancer cells.

### 3.4. CXCL11 Rescued the Suppression of the Migration and Invasion in Cervical Cancer Cells by the EphA2 Knockout

**Figure 5:**
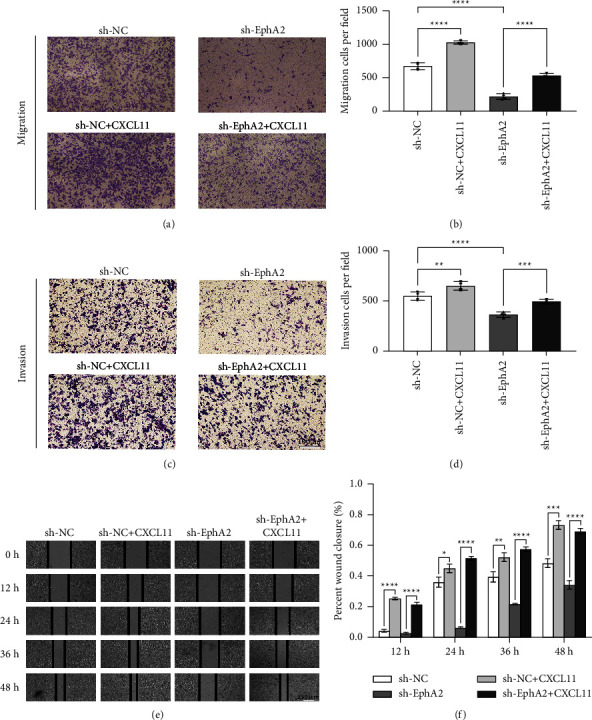
Exogenous CXCL11 supplementation rescues the inhibitory effect of EphA2 on cervical cancer migration and invasion ability. (a)-(b) The effect of CXCL11 on the migration ability of cervical cancer cells after EphA2 knockdown. (c)-(d) The effect of CXCL11 on the invasive ability of cervical cancer cells after EphA2 knockdown. (e)-(f) Wound healing assay results indicating that CXCL11 rescued the impaired migration ability of cervical cancer cells with low EphA2 expression.^*∗*^*p* < 0.05; ^*∗∗*^*p* < 0.01; ^*∗∗∗*^*p* < 0.001.

We assessed the effect of CXCL11 on the metastatic ability of sh-EphA2-SiHa cells by a transwell migration and invasion assay and the wound healing assay. The results of the transwell migration and invasion assay and the wound healing assay together demonstrated that the addition of CXCL11 rescued the inhibition of cervical carcinoma cell migration and invasion by the EphA2 knockout. Meanwhile, CXCL11 also played a role in promoting invasion and invasive ability in normal SiHa cells (Figures 5(a)–5(f)).

### 3.5. PD-L1 Mediates the Oncogenic Role of EphA2-CXCL11 in Cervical Cancer

**Figure 6:**
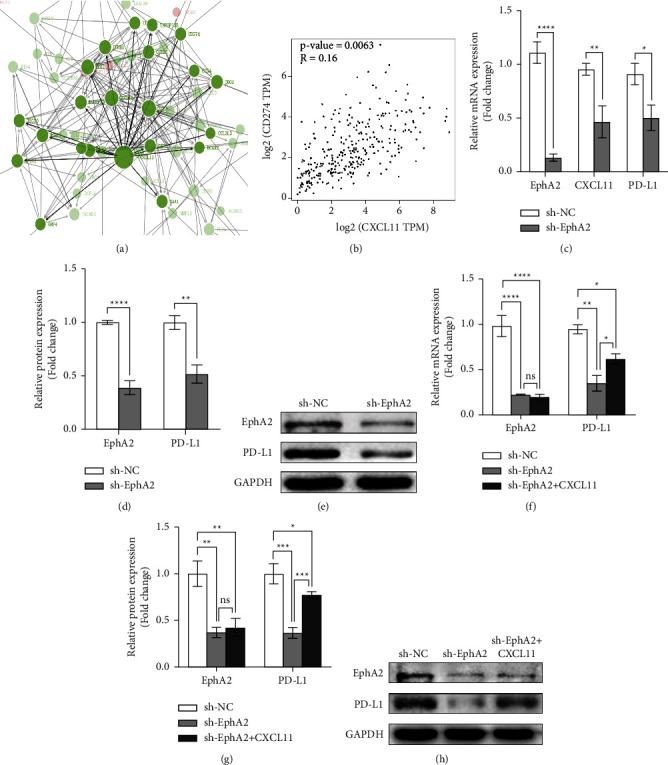
EphA2 further regulates PD-L1 expression through CXCL11. (a) Protein interaction map from sequencing analysis, indicating protein interaction between CXCL11 and PD-L1. (b) Correlation of PD-L1 and CXCL11 in cervical cancer in GEPIA database. (c) Relative messenger RNA expression of CXCL11 and PD-L1 after EphA2 was knocked down. (d)-(e) Relative expression and gray value of PD-L1 after EphA2 was knocked down. (f)–(h) Replenishment of CXCL11 rescues PD-L1 expression at messenger RNA and protein levels. ^*∗*^*p* < 0.05; ^*∗∗*^*p* < 0.01; ^*∗∗∗*^*p* < 0.001.

To further study the molecular mechanism of EphA2 in cervical cancer, we explored the downstream molecules of EphA2/CXCL11. We found that the protein interaction map in the sequencing analysis suggested a protein interaction between CXCL11 and PD-L1 (Figure 6(a)). Meanwhile, the GEPIA database showed that PD-L1 and CXCL11 were highly correlated in cervical cancer (*p*=0.0063) (Figure 6(b)).

From this finding, we speculate that PD-L1 mediates the cancer-promoting effect of EphA2-CXCL11 in cervical cancer. PD-L1 is considered a cosuppressor of immune responses. As a tumor-promoting factor, it has a critical role in tumor immune escape by attenuating the host's immune response to tumor cells by binding to its receptor [20]. In addition, PD-L1 exerts nonimmunoproliferative effects on various types of tumor cells. PD-L1 in tumor cells has a key role in promoting cancer stemness, epithelial-mesenchymal transition, tumor invasion, and chemoresistance in multiple solid tumors [21]. Studies have shown that highly expressed PD-L1 in cervical carcinoma cells can promote the growth and metastasis of cervical cancer through the ITGB4/SNAI1/SIRT3 pathway [22]. The expression of PD-L1 is regulated by many factors [23], among which a variety of cytokines play an important role in regulating the expression of PD-L1, which induces the upregulation of PD-L1 in tumor cells through the tumor microenvironment [24–26].

Existing studies have demonstrated that tumor cells can create a tumor-promoting microenvironment through autocrine cytokines and chemokines, and this inflammatory environment can promote cancer cell survival and growth [27–30]. Moreover, it has been reported in the literature that tumor cells affect the expression of PD-L1 in cells by autocrine CXCL11 [31, 32], so we have reason to believe that EphA2 upregulates PD-L1 through autocrine chemokine CXCL11, thereby promoting the occurrence and development of cervical carcinoma. To verify the authenticity of this speculation, we detected changes in PD-L1 expression levels in the sh-EphA2-SiHa cell line by qRT-PCR and Western blotting, and the detection results were consistent with our speculation (Figures 6(c)–6(e)). Then, we verified the regulatory effect of CXCL11 on PD-L1. By examining the effect of CXCL11 on the expression level of PD-L1 in sh-EphA2-SiHa cells, we found that CXCL11 increased the expression of PD-L1 at both the messenger RNA and protein levels (Figures 6(f)–6(h)). Overall, we have reason to believe that EphA2 promotes the development of cervical cancer through the CXCL11/PD-L1 pathway.

### 3.6. EphA2 Regulates the Expression of CXCL11 and PD-L1 In Vivo

**Figure 7:**
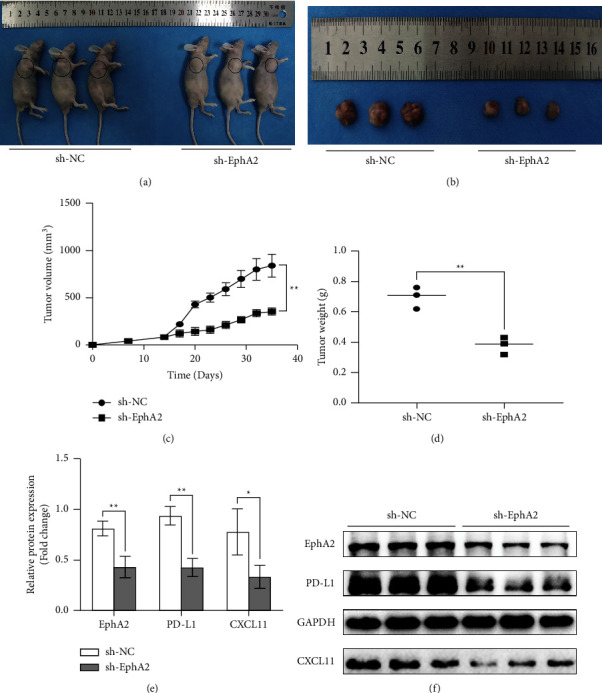
Inhibition of EphA2 decreases the expression of CXCL11 and PD-L1 in the xenograft study. (a) Photos taken immediately after the nude mice were humanely sacrificed. (b) Photos of tumor samples taken from nude mice in each group. (c) Tumor volume measured at 1 week, 2 weeks, and every 3 days after 2 weeks. (d) The weight of tumors in the sh-EphA2 group and sh-NC group. (e)-(f) Relative expression and gray value of CXCL11 and PD-L1 proteins after EphA2 inhibition in vivo. ^*∗*^*p* < 0.05; ^*∗∗*^*p* < 0.01; ^*∗∗∗*^*p* < 0.001.

To investigate whether EphA2 can also regulate the expression of CXCL11 and PD-L1 in vivo, we established the xenograft cervical tumor model. According to the monitoring results of tumor volume and weight, we found that the volume and weight of the tumor after the EphA2 knockdown were lower than those of the control group (Figures 7(a)–7(d)). A Western blot analysis of the removed tumor tissue showed that the expression of CXCL11 and PD-L1 decreased synchronously after EphA2 was knocked down (Figures 7(e) and 7(f)). This indicates that EphA2 also has a regulatory effect on CXCL11/PD-L1 in vivo, further proving the possibility of the existence of the EphA2/CXCL11/PD-L1 pathway.

## 4. Discussion

Cervical carcinoma is the most common gynecological malignancy, and its mortality rate ranks first among all gynecological malignancies [1]. The threat of cervical carcinoma to a woman's life and health prompted us to seek more and better treatment methods, and EphA2 should receive more attention as a potential therapeutic target in cervical carcinoma. Our previous research has proved the cancer-promoting activity of EphA2 in cervical cancer [12], which was further confirmed in this study. The tumor biological functions of cervical cancer cells were decreased with EphA2 knockdown, whether in proliferation, invasion, or metastasis.

Some studies have reported the carcinogenic mechanism of EphA2 in some malignant tumors [33–35]. However, the molecular mechanism of EphA2 in promoting cervical cancer has not been studied. To gain a more comprehensive understanding of the molecular mechanisms by which EphA2 exerts the tumor-promoting effect in cervical cancer, we performed an RNA sequencing analysis of EphA2 knockdown SiHa cells and SiHa cells that originally overexpressed EphA2. According to the sequencing results, we discovered the downstream factor of EphA2 and CXCL11 and verified the regulatory effect of EphA2 on CXCL11 through related experiments. In cervical cancer cells, CXCL11 was highly expressed when EphA2 was highly expressed. Knockdown of EphA2 results in a decrease in the expression level of CXCL11. Regarding the cancer-promoting effect of CXCL11, existing research has shown that CXCL11 can directly affect the proliferation and migration of tumor cells or indirectly control tumor angiogenesis and regulate leukocyte infiltration to control tumor growth, metastasis, and lymphatic infiltration [13–16, 36]. This outcome is consistent with our experimental results. As a chemotactic cytokine, CXCL11 is overexpressed in various solid tumors and promotes tumorigenesis and development, but little research has been conducted in cervical cancer. Our findings not only demonstrate the promoting effect of CXCL11 on the proliferation, invasion, and metastasis of cervical carcinoma but also the mediation of CXCL11 on the cancer-promoting effect of EphA2.

Subsequently, we found a possible relationship between CXCL11 and PD-L1 through the protein interaction map in the sequencing analysis results; the GEPIA database also suggested a correlation between CXCL11 and PD-L1. In addition, some studies have demonstrated the regulatory effect of autocrine CXCL11 on PD-L1 by tumor cells [31, 32]. As a recognized immune cosuppressive molecule, PD-L1 attenuates the host's immune response to tumor cells by transmitting inhibitory signals, promotes the immune escape of tumor cells, and plays a cancer-promoting role in various types of malignant tumors [37–39]. Therefore, we speculate that there is a signal transduction pathway of EphA2/CXCL11/PD-L1 in cervical cancer. Relevant experimental results showed that the knockdown of EphA2 led to the low expression of PD-L1, and replenishment of CXCL11 reversed this trend, resulting in a recovery of PD-L1 expression levels. This finding indicates that PD-L1 is likely to play a tumor-promoting role as a downstream molecule of EphA2/CXCL11. To further confirm the possibility of the existence of this pathway, we established an in vivo mouse model of cervical cancer by subcutaneous tumorigenesis. The measurement data showed that knockdown of EphA2 significantly inhibited tumor growth. After the mice were humanely killed, we performed a Western blot analysis on the isolated tumors. The experimental data showed that the expressions of CXCL11 and PD-L1 in the sh-EphA2 group were significantly inhibited compared with the control group. This finding gives us all more reason to believe that EphA2 promotes the development of cervical cancer through the CXCL11/PD-L1 pathway.

Although our research shows the existence of the EphA2/CXCL11/PD-L1 pathway, we do not deny the existence of other complex EphA2-related signaling pathways. Existing studies have reported a variety of cancer-promoting mechanisms related to EphA2 [35, 40, 41]. As a transmembrane protein with tyrosine kinase activity, EphA2 can activate downstream signaling when phosphorylated [42]. We believe that EphA2 activation increases the expression of CXCL11 through a series of complex signaling processes. CXCL11 then upregulates PD-L1 expression signaling by binding to CXCR3 and activating the STAT and PI3K-Akt signaling pathways, as reported in previous studies [32]. Of course, this is only speculation based on existing research. More studies are needed to verify this hypothesis and better understand the mechanism of EphA2 in cervical cancer.

The important role of PD-L1 in multiple cancers makes it a therapeutic target for many malignant tumors, and inhibitors targeting PD-L1 have been approved for the treatment of certain cancers [43–45]. Previous studies have shown that PD-L1 is overexpressed in cervical cancer and promotes its growth and metastasis [46], which is an important biomarker for evaluating the prognosis and clinicopathological features of cervical cancer [47]. In clinical trials related to patients with cervical cancer, the effectiveness and safety of durvalumab were assessed in combination with radiotherapy, ADXS11-001, tremelimumab, and Vigil (NCT03452332, NCT02291055, NCT01975831, and NCT02725489). Immunotherapy against PD-L1 has become a research hotspot, and a variety of PD-L1 monoclonal antibodies have been put into clinical use and achieved certain curative effects. The study of PD-L1-related signaling pathways will help us to better understand the molecular mechanisms of PD-L1 on cancer, thereby providing new guidance for immunotherapy. This study proposes the EphA2/CXCL11/PD-L1 signaling pathway for the first time, providing a new option for PD-L1-related immunotherapy and combination therapy. We speculate that targeting EphA2 combined with targeting PD-L1 can produce better efficacy than a single drug, that is, targeting EphA2 may enhance the sensitivity of cervical cancer to PD-L1-targeted therapy. For patients who are insensitive to or resistant to PD-L1-targeted therapy, targeting EphA2 in combination with targeting PD-L1 may achieve greater benefits. However, more research and clinical trials are needed to confirm this speculation.

**Figure 8:**
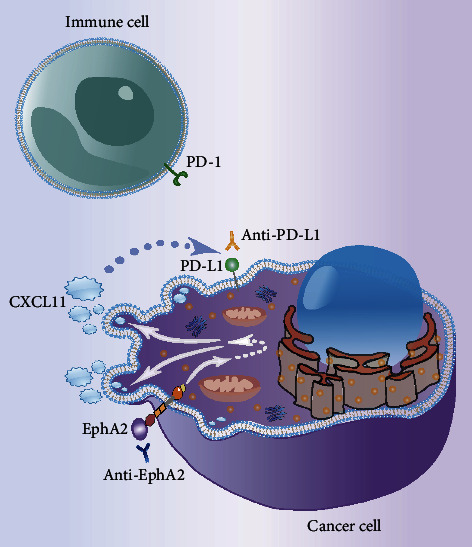
Schematic representation of EphA2 promoting cancer through the CXCL11/PD-L1 pathway. The dotted line indicates that there may be other signal transduction mechanisms between the two molecules.

This study proposes and validates the EphA2/CXCL11/PD-L1 signaling pathway. It is believed that EphA2 affects the tumor microenvironment by promoting the autocrine chemokine CXCL11 in cervical cancer cells and further regulates the expression of PD-L1 on the cell surface. Combination therapy of anti-EphA2 and anti-PD-L1 may produce a better tumor suppressor effect. Given the diversity and complexity of cell signaling pathways, there may be multiple EphA2-related signaling pathways in tumor cells, which together play a role in promoting cancer (Figure 8). To achieve the better clinical therapeutic effect by targeting EphA2, a more comprehensive understanding of the cancer-promoting mechanism of EphA2 is required.

## 5. Conclusions

This article explores the molecular mechanism of the cancer-promoting role of EphA2 in cervical cancer, and the results show that EphA2 promotes the proliferation, invasion, and metastasis of cervical cancer through the CXCL11/PD-L1 pathway. Collectively, our study uncovered a possible cancer-promoting mechanism of EphA2 and proposed for the first time that EphA2 promotes the development of cervical cancer through the CXCL11/PD-L1 pathway. This finding provides new ideas for future research as well as the targeted therapy and combined therapy for cervical cancer.

## Figures and Tables

**Table 1:** Gene primer sequence (5′-3′).

Gene	Primer sequence (5′-3′)
EphA2	Forward: 5′-TGGCTCACACACCCGTATG-3′
	Reverse: 5′-GTCGCCAGACATCACGTTG-3′

CXCL11	Forward: 5′-GACGCTGTCTTTGCATAGGC-3′
	Reverse: 5′-GGATTTAGGCATCGTTGTCCTTT-3′

PD-L1	Forward: 5′-CTGGACAAGCAGTGACCATCAA-3′
	Reverse: 5′-TCAGTGCTACACCAAGGCATAAT-3′

GAPDH	Forward: 5′-CTTAGTTGCGTTACACCCTTTCTTG-3′
	Reverse: 5′-CTGTCACCTTCACCGTTCCAGTTT-3′

**Table 2:** Corresponding primary antibody.

Gene	Primary antibody
EphA2	66736-1-Ig, mouse antihuman, 1 : 1,0000, Proteintech
CXCL11	DF9917, rabbit antihuman, 1 : 500, Affinity
PD-L1	66248-1-Ig, mouse antihuman, 1 : 5,000, Proteintech
GAPDH	bs2188R, rabbit antihuman, 1 : 5,000, Bioss

## Data Availability

The datasets generated during and/or analyzed during the current study are available from the corresponding author upon request. The relevant sequencing results have been submitted to the National Center for Biotechnology Information Sequence Read Archive (https://www.ncbi.nlm.nih.gov/sra).
